# Dynamic survival prediction in intensive care units from heterogeneous time series without the need for variable selection or curation

**DOI:** 10.1038/s41598-020-79142-z

**Published:** 2020-12-17

**Authors:** Jacob Deasy, Pietro Liò, Ari Ercole

**Affiliations:** 1 Computer Laboratory, University of Cambridge, William Gates Building, 15 JJ Thomson Ave, Cambridge, CB3 0FD UK; 2 Division of Anaesthesia, Addenbrooke’s Hospital, University of Cambridge, Hills Road, Cambridge, CB2 0QQ UK

**Keywords:** Outcomes research, Health care, Medical research

## Abstract

Extensive monitoring in intensive care units (ICUs) generates large quantities of data which contain numerous trends that are difficult for clinicians to systematically evaluate. Current approaches to such heterogeneity in electronic health records (EHRs) discard pertinent information. We present a deep learning pipeline that uses all uncurated chart, lab, and output events for prediction of in-hospital mortality without variable selection. Over 21,000 ICU patients and tens of thousands of variables derived from the MIMIC-III database were used to train and validate our model. Recordings in the first few hours of a patient’s stay were found to be strongly predictive of mortality, outperforming models using SAPS II and OASIS scores, AUROC 0.72 and 0.76 at 24 h respectively, within just 12 h of ICU admission. Our model achieves a very strong predictive performance of AUROC 0.85 (95% CI 0.83–0.86) after 48 h. Predictive performance increases over the first 48 h, but suffers from diminishing returns, providing rationale for time-limited trials of critical care and suggesting that the timing of decision making can be optimised and individualised.

## Introduction

Clinicians in the intensive care unit (ICU) frequently need to make outcome- and time-critical decisions. To this end, ICU patients are routinely highly investigated and monitored, providing data to alert health care providers to deterioration and optimally inform decision making. As a result, the ICU has higher data volume, variety, and velocity than any other clinical setting. Such considerations make it a challenge to fully appreciate all of the information available, as well as temporal relationships between clinical variables, particularly in the context of complex antecedent events and disease histories in a dynamically evolving environment.

On a day-to-day basis, it is unlikely that clinicians fully, systematically, and robustly appraise all the information routinely available to them. For example, the fact that it is possible for algorithms with less data to outperform clinicians when considering which ICU patients can successfully be stepped down^1^, suggests that not all predictive power available is exploited in decision making. At best, this could represent a missed opportunity to improve care. At worst, information-overload may compromise patient safety, since human factors research has demonstrated that being overwhelmed by data leads to unconscious, and therefore potentially sub-optimal, exclusion of available information to once again make rapid decision making tractable (as is well illustrated in the ICU by the phenomenon of alarm fatigue^2,3^). Such considerations suggest that critical care is an area that is highly likely to benefit from successful automated exploitation of EHRs to assist clinicians in making optimally informed decisions^4,5^.

However, any technology to help the clinician must deal with data that is highly heterogeneous, both in type (ranging from continuous variables such as laboratory results to event data such as interventions, drug administrations, or clinical assessments) and in sampling rate (which ranges from demographic parameters to time-series data). Furthermore, the data may be subject to variable or irregular sampling and possibly informative missingness^6^. A final challenge is that ICU admissions are dynamic—prognostic accuracy is not static but changes over time^7^. Clinicians, and any supportive modelling system, must continually reconsider prognosis likelihoods while attending to the readings of multiple patients, each of whom may have a vast array of differing predictor variables^8–11^.

Rajkomar et al. presented the first attempt to use all available patient data by mapping the entire EHR to a highly curated set of predictor variables, structured inline with the categories of data available^12^. Although this method achieved strong performance, risk assessment was EHR format-specific, static, and reliant on an ensemble of diverse model structures. We conjecture that the embedding mechanism outlined therein could be generalised to the higher resolution setting of the ICU to usefully provide a dynamic estimate of survival probability as a composite surrogate for patient state. We extend this method to make it time sensitive; allowing predictions to be made at arbitrary points, optimally utilising all information available at the time. Our objective was to design and implement a pipeline for prediction which incorporated all chart, lab, and output events in the same way, without the need for variable selection or manual curation. Furthermore, we sought to evaluate model explainability by ranking the features the model attended to when making its predictions.

### Background

In the United States, over a third of hospitals now utilise EHR databases that are considered broad enough to be ‘comprehensive’^13^. These extensive EHRs have shown promise as cohorts for retrospective studies, avoiding many of the ethical and procedural difficulties of randomised control trials . However, a systematic review of 107 predictive models built with EHR data^4^ found that only 34$\cdot$6% (37/107) used longitudinal data and the median number of variables used was only 27. In the ICU, clinicians tend to make use of an even smaller set of variables to categorise patients based on long-standing severity scales^14,15^. Mortality risk estimates are often based on acute physiology scores for disease severity—such as the Simplified Acute Physiology Score II^16^ (SAPS II) and the Acute Physiologic Assessment and Chronic Health Evaluation^17^ (APACHE). These metrics are static and solely based on logistic regression of specific markers of patient physiology that are recorded during the first hours after ICU admission.

Over the past few years, the deep learning literature indicates that models such as Recurrent Neural Networks^18,19^ (RNNs), such as Long Short-Term Memory (LSTM) networks^20^, have been shown to outperform these traditional models^5^. However, the dominant approach to deep learning with EHR data remains reliant on an initial variable selection stage, involving the use of expert knowledge to hand-select a subset of clinically relevant variables^6,21^ This is time-consuming and suitably predictive variables may not always generalise between datasets: They may either not be consistently recorded or may differ in their definitions. Therefore, a method for generating predictions without the need for this step would be an important advance in creating systems that could be deployed and trained locally, taking into account all available information without specialist feature-engineering. Furthermore, any system attempting to use real world data must be robust to the shortcomings of routinely collected data such as missingness, data entry errors, and implausible values, as well as undesirable statistical distribution properties. A common approach to this problem is to first *curate* the data: a slow process where the data quality is first assessed by hand according to data definitions and corrective action taken where necessary. However, this can be a painstaking manual task which does not scale to very large sets of predictive features. Developing a system which obviates the need for such a delay would be highly advantageous in terms of local training and deployment. Attempts to incorporate a broader set of variables have used pretrained word embeddings from outside of the medical context and have avoided numerical values^22^. In the literature, we have found no examples of models which perform zero variable selection, data processing, or model ensembling. Therefore, the development of a flexible deep learning pipeline and model which incorporates broad patient data could potentially provide a more versatile and accurate outcome prediction method in the ICU.

In the clinic, many extraneous clinical variables are recorded but seldom utilized to inform clinical decision making. To our knowledge, our study is the first in which all patient chart, lab, and output events have been aggregated in the same way to dynamically predict the risk of mortality for ICU patients. We used records from more than 20,000 ICU patients with nearly 5000 unique event types from the MIMIC-III dataset. Models based on broad EHR data could predict mortality far better than the clinical severity scores currently in use. Aggregation of all event types in a RNN showed that health-related information from varied time points and clinical processes interact in a non-linear manner. Additionally, taking account of these interactions between rarely-related clinical variables gave more precise prognostic estimates than simply using acute physiology measures.

Our study demonstrates the importance of exploiting a broad range of clinical variables when predicting the risk of mortality amongst ICU patients, and thus, the importance of these data in clinical decision making. The predictive power of the additional clinical variables in our model leads to interpretable and more personalised dynamic prediction, requires no manual data curation or variable selection, and provides reliable performance after only a few hours in the ICU.

## Methods

### Data sources

We built and validated a computational clinical support model using retrospective analysis of adult patient data using the MIMIC-III database. The database contains high-resolution patient data, including: demographics, vital-sign time-series, laboratory tests, medications and procedures, fluid intake and outputs, clinician notes, and diagnostic coding. The demographic characteristics of this dataset have been previously described^23^.

### Procedures

**Figure 1:**
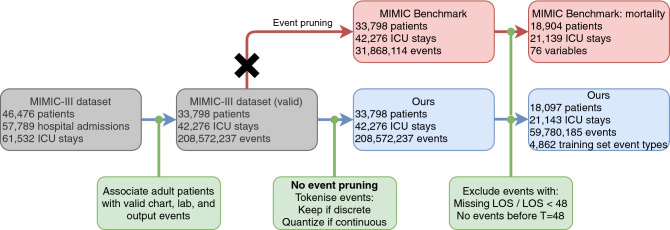
Patient and event summary statistics at each stage of our pipeline (blue) compared to the MIMIC-III benchmark (red). We replace event pruning with tokenisation, which leads to a broader set of variables making up a larger set of events. For one training split, there are 4862 event types, 3678 of which are continuous, leading to 31,913 possible variables when 10 percentile bins are used. In this diagram, ‘MIMIC Benchmark’ refers to the processing in Harutyunyan el al.^21^.

**Figure 2:**
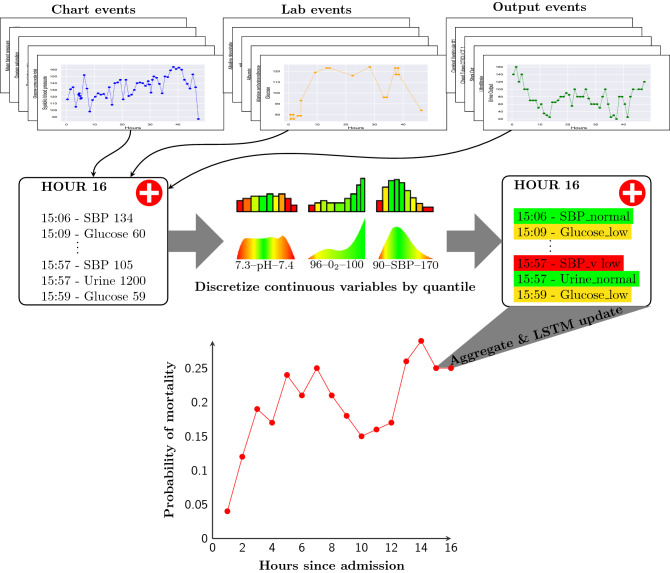
Diagram of our model. For each patient, all chart, lab, and output events are assembled into fixed time periods. Continuous data, such as Systolic Blood Pressure, Glucose, and Urine Output, are discretized into quantile-based bins, allowing continuous features to be mapped to an embedding. Discrete chart and lab events, from a diverse range of investigations, are equivalently aggregated by the model. Both continuous and discrete events from 1 h of patient timeseries data are embedded and aggregated, using a learnt variable importance ranking (colour in central distributions). The weighted average embedding is then used as input to a LSTM recurrent neural network which generates an updated dynamic prediction of in-hospital mortality probability each hour and updates an internal representation of the patient state. Dynamic prediction allows for continual patient monitoring as new data is accumulated and used to update outcome probabilities. Laboratory values, physiological readings, and admission information was from the first 48 h after ICU admission. *LSTM* long short-term memory.

Unlike traditional approaches, we retain all of the chart, lab, and output events for each stay without any data cleaning, outlier removal, or domain-specific knowledge. We perform the necessary assignment of a patient, a stay-ID, and a timestamp to each event—a process which is independent of EHR data formatting or structure. Our model then uses the entire patient timeseries for a given window (inline with literature on mortality prediction^12,21^, this is chosen to be 48 h) as input, regardless of event type, frequency, or cardinality. As outlined in Figs. 1 and 2, because we do not select for clinical variables, after event association with patient stays, the set of full timeseries in our EHR dataset contains 208,572,237 events instead of the 31,868,114 employed in the MIMIC-II benchmark^21^ and all subsequent papers relying on this starting point. After truncation of patient stays to 48 h worth of data, our data-independent pipeline still contain 59,780,185 events. The increased number of variables used by our model also means marginally more patients and stays are retained, supporting the evidence in favour of models which can incorporate broad EHR data and minimise patient exclusion based on obscure EHR recordings.

To distinguish between discrete and continuous variables, we label events by whether their values can be converted to a floating point number. This captures all integer or decimal events, such as heart rate or blood pH, and ignores discrete labels (E.g. ‘Code Status Full Code’). Additionally, unusual or faulty cases, such as readings with multiple decimal places are designated as discrete, making our model robust to and aware of consistent errors which potentially correlate with patient outcomes. Unlike prior work^22^, we make use of missing and numerical values. Missing readings are considered as separate discrete events so that our model can capitalise on ‘informative missingness’^6,24^. Whereas, we tokenize continuous values by quantizing them into discrete bins by percentile—our default model uses ten such bins. Multiple quantiles reinforce the robust nature of our model, as any outliers (e.g. a regular mistake in blood pH data is for a faulty machine to record a pH of 5.5) are likely to be contained in tokens at the periphery of a variable’s distribution, and the model can learn to ignore these extreme tokens. Examples of this tokenization and quantization procedure are given in Table 1 and Equation 1.

**Table 1:** Examples of discrete token creation alongside percentile-based quantization and tokenization of continuous variables.

Label	Value	Token
Eye opening	4 Spontaneously	Eye Opening_4 Spontaneously
Heart rate	84	Heart Rate_8
Code status	Full code	Code Status_Full Code
Systolic blood pressure	NaN	Systolic blood pressure_NaN

Discrete variables remain discrete, while values that can be converted to floating point numbers are considered continuous and separated into percentile-based bins. Our pipeline is simply: *convert non-NaN numeric values to percentiles, everything else is considered discrete.*

### Model development

Patient data were considered as a multivariate timeseries defined by: the times when patient events were recorded, the sequence of indexes mapped from each patient’s discretized timeseries, and the set of outcomes for each patient episode. Patient survival was labelled as one, and patient death as zero. Our baseline model used snap shots that contained all chart, lab, and output events for each hour of a patient’s timeseries. RNN models were trained on chart, lab, and output data that met the inclusion criteria within the chosen time period. A ten-fold cross-validation scheme was used to prevent overfitting, in addition to an independent test set. Data for ICU admissions were split into a training set (90%) and an independent test set (10%). For each cross-validation fold, training data were divided into a training set and a 1000 patient validation set, with the split stratified by survival to ensure balanced training. A Long Short-Term Memory (LSTM) RNN architecture of depth one, with a single output head per time step, was trained by backpropagation on the training set. As the patient-specific nature of EHR data is prone to overfitting, training was stopped once the validation set AUROC plateaued for more than five epochs. During training, model performance on the validation set was continually assessed, and the optimal model for each cross-validation fold was selected from the epoch with the best validation set performance.

As EHR data is known to be broad and highly variable^5^, even after all continuous variables in the relatively small MIMIC-III database were binned into ten discrete percentile categories, the number of variables remaining was still 31,913. Therefore, traditional methods, such as learning a transition dynamics matrix or one-hot encoding all of the input variables, would be prohibitively expensive and likely to overfit. Therefore, to circumvent this potential over-parameterization, our model takes inspiration from natural language processing^25,26^, where input and output vocabularies for translation are often very large. In the case of many thousands of medical tokens it is more computationally efficient to let the model learn a common low-dimensional vector representation of each token.

We map tokens recorded in the model’s medical vocabulary according to

1 $$\begin{aligned} \overbrace{ \begin{bmatrix} \text {Eye Opening 4 Spontaneously}\\ \text {Heart Rate}\_\text {8}\\ \text {Code Status Full Code}\\ \vdots \end{bmatrix} }^{\text {Discrete event tokens in 1 h}} \rightarrow \overbrace{ \begin{bmatrix} {-0.22,\, 0.34,\ldots ,\, 0.83} \\ {0.52,\, 0.03,\ldots ,\, -0.28} \\ {-0.21,\, 0.17,\ldots ,\, 0.89} \\ \vdots \end{bmatrix} }^{\text {Corresponding learnt vectors}}. \end{aligned}$$

The size of the embedding vectors was optimized via grid search over values 16, 32, 48, and 64. Embedding dropout^27,28^ was applied to regularize the network and prevent the model from overfitting to strongly predictive tokens which may not be available for all patients. For each patient, we allow a maximum of 5000 events over the initial 48 h of their stay in the ICU. For the few patients who have more than 5000 events, we extract their final 5000 tokens. We tested using an average vector per time period, vectors for both tokens and time period, and aggregating vectors with learnt weights. The final method performed on par with the first two, but has the added advantage of producing a ranked list of variable importance after model training—beneficial for understanding what information the model is prioritising. Therefore, we aggregate each patient timeseries snapshot according to

2 $$\begin{aligned} \overbrace{ \begin{bmatrix} w_{0}, w_{1}, w_{3}, \ldots \end{bmatrix} }^{\text {Learnt weights}} \overbrace{ \begin{bmatrix} {-0.22,\, 0.34,\ldots ,\, 0.83} \\ {0.52,\, 0.03,\ldots ,\, -0.28} \\ {-0.21,\, 0.17,\ldots ,\, 0.89}\\ \vdots \end{bmatrix} }^{\text {Learnt vectors}}&= \overbrace{ \begin{bmatrix} {0.04,-0.52,\ldots ,-0.72} \end{bmatrix} }^{\text {Aggregated hourly vector}}, \end{aligned}$$

and pass this as input to a long short-term memory (LSTM) RNN^18,19^.

Finally, we use a densely connected layer with a sigmoid activation function to output $p(y_{i}|X_{i})\in [0,1]$, the probability of in-patient mortality given a patient timeseries, and optimize the parameters of our model by minimising the binary cross entropy loss

3 $$\begin{aligned} {\mathscr {L}}(y, \tilde{y}) = -\sum \limits _{i=1}^{N} \sum \limits _{t=0}^{T} \overbrace{\tilde{y}_{it}\log y_{i}}^{\text {Misclassified death loss}} + \overbrace{(1-\tilde{y}_{it})\log (1-y_{i})}^{\text {Misclassified survival loss}}, \end{aligned}$$

across each training batch and through time. The Adam optimizer^29^ was used for training; output activation function (sigmoid), batch size (128), and learning rate (0.0005) were kept constant across models. The number of hidden neurons (32, 64, 128, and 256 units), as well as the probability of embedding drop-out were optimized via a grid search over the models. To establish confidence intervals, we used the bootstrap algorithm^30^ with 10,000 samples of the test set performance using the cross-validated models. A model summary is presented in Fig. 2.

## Results

The combined MIMIC-III CareVue and MetaVision dataset consisted of 330,712,483 chart events, 27,854,055 lab events, and 4,349,218 output events from 46,476 patients with 61,532 ICU stays. In the cohort we use for mortality prediction, the percentage of stays with in-hospital deaths was 13.2% (2797/21,143) and the proportion of long ICU stays (more than seven days in the ICU) was 23.0% (4672/21,143). The mean and median length of stay in the ICU was 5.97 and 3.72 days respectively. During the first 48 h in the ICU, an average admission had 1268 events, drawn from a possible 2353 unique event names. The data were split into training and test sets which had chart, lab, and output events available.

**Table 2:** Statistics for our subset of the MIMIC-III dataset used for model training, validation, and testing before truncation to 48 h.

Variable	Count	$Q_1$	$Q_2$	$Q_3$	Unit of measurement
Anion gap	431,485	11.00	13.00	15.00	mEq/L
Albumin	68,632	2.50	3.00	3.50	g/L
Bicarbonate	438,884	22.00	25.00	28.00	mEq/L
Bilirubin	104,367	0.40	0.90	2.40	mg/dL
Creatinine	450,734	0.70	1.00	1.60	mg/dL
Chloride	481,780	100.00	104.00	108.00	mEq/L
Diastolic BP	4,166,196	50.00	59.00	69.00	mmHg
GCS Total	641,931	9.00	13.00	15.00	
Glucose	568,905	100.00	121.00	150.00	mmol/L
Heart rate	4,262,499	73.00	85.00	98.00	/min
Hematocrit	560,500	27.10	30.10	33.90	% of blood volume
Hemoglobin	477,317	9.10	10.20	11.50	g/L
FiO2	372,891	40.00	40.00	50.00	%
Lactate	126,034	1.20	1.80	3.00	mmol/L
Mean ABP	4,208,706	68.00	77.00	89.00	mmHg
Platelet	428,028	137.00	210.00	301.00	1000/mm$^3$
Potassium	608,058	3.70	4.10	4.40	mEq/L
PTT	286,946	28.00	34.00	51.10	s
PT	270,192	13.10	14.40	17.10	s
Sodium	506,194	136.00	139.00	141.00	mEq/L
Oxygen saturation	4,151,164	96.00	98.00	99.00	%
Respiratory rate	4,707,473	16.00	19.00	24.00	/min
Systolic BP	4,179,045	104.00	119.00	137.00	mmHg
Temperature (F)	892,931	97.60	98.50	99.50	$^{\circ }$F
BUN	448,975	14.00	21.00	36.00	mg/dL
WBC	414,369	7.00	9.70	13.40	1000/mm$^3$

We present the lower quartile, median, and upper quartile values ($Q_{1}, Q_{2}$, and $Q_{3}$ respectively) as, unlike mean and standard deviation, they are not skewed by the outliers deliberately kept in our dataset.

**Figure 3:**
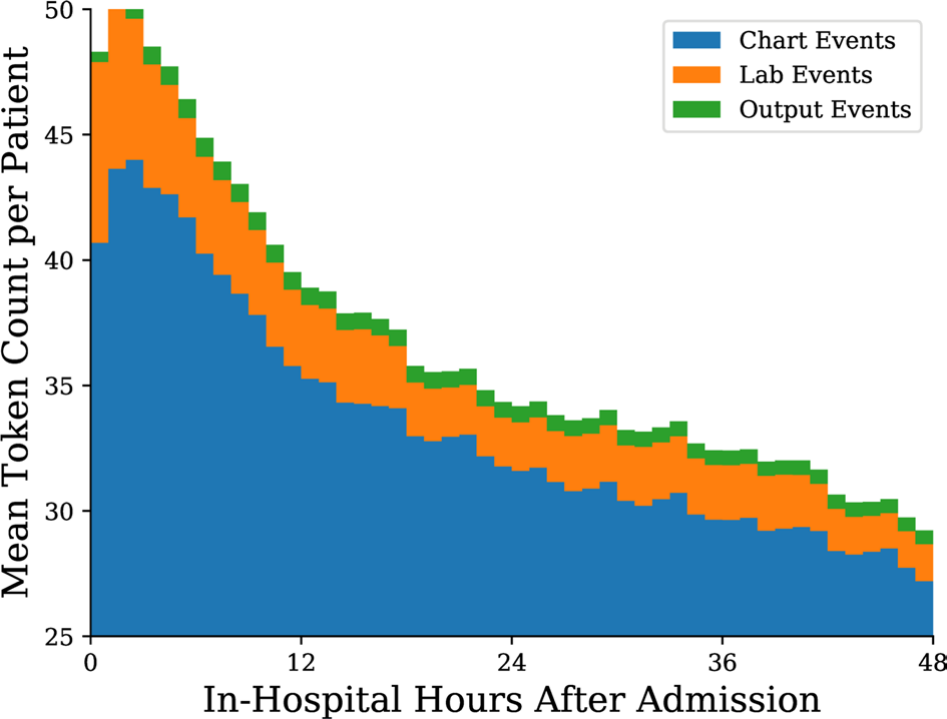
Distribution of mean token count per patient per hour, stratified by type, for the first 48 h since ICU admission for those patients whose stay was at least that long.

Patient event types over time are summarised in Fig. 3 and summary statistics are provided in Table 2. In the first few hours after admission, the average patient has 45–50 readings per hour. Despite the majority of events in any given hour being chart events, in the first few hours there are also additional lab events as initial patient data is accumulated. After the initial peak in readings per hour, the number of chart events recorded declines quickly over the first 12 h, before continuing to decline in a slower fashion for the remainder of each patient’s stay. Finally, towards the 48 h mark patient events are predominantly comprised of chart events, with an average rate of less than 30 tokens per hour.

**Figure 4:**
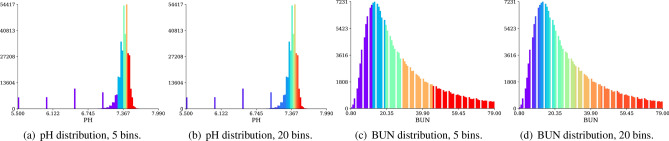
Representative examples of the distribution of blood pH and blood urea nitrogen (BUN) illustrating the effect of discretisation by ‘binning’. In our method, continuous variables are discretised by distribution frequency so that all data types can be handled in the same way in the model. Colours exemplify 5 or 20 discrete categories established by our pipeline for any continuous variable, demonstrating outlier category detection and the increased granularity in populous intervals found by percentile-based quantization. For BUN the distribution is fairly continuous and binning creates a representation which naturally encodes the concepts of ‘high’ or ‘low’ within the distribution. For variables such as pH however, the discretisation also places artefactual values into one (**a**) or more (**b**) ‘outlier’ bins. If artefacts are random, the model should be able to learn that such data points have no predictive value and can therefore be ignored.

The outcomes of our discretization pipeline for two variables, blood pH (PH) and blood urea nitrogen (BUN), are displayed in Fig. 4. When employing five percentile-based bins in Fig. 4a, our pipeline successfully discarded several tens of thousands of outlier readings which are the result of technical faults in recording equipment as they are not physiologically possible (e.g. blood pH below 7). Further dividing pH values into twenty percentile-based bins in Fig. 4b, it is even possible to distinguish between different outlier categories—each of which may have a slightly different effect on patient outcomes. In the case of blood urea nitrogen, values are distributed more uniformly, with fewer outliers, so the main advantage of value percentile-based discretization lies in differentiating between populous patient categories towards the distribution’s centre of mass.

### Model comparison

**Figure 5:**
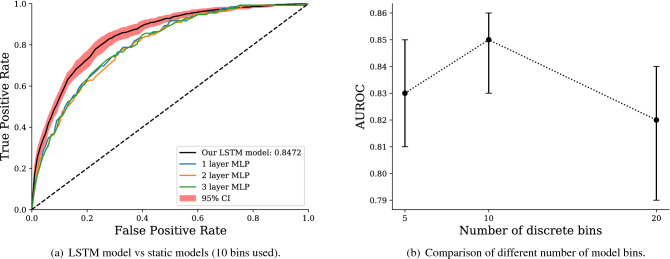
Model variant comparison. The left panel demonstrates that employing the recurrent LSTM architecture after the model embedding layer leads to a significant increase in model performance. The right panel depicts the impact of differing the number of continuous value percentile bins. When considering 5, 10, and 20 bins, we find that 10 offers the best performance and use this in all other results. 95% Confidence intervals are bootstrapped from a 10-fold cross-validation using 10,000 resamples.

We compare different variants of our model in Fig. 5a. We contrast our LSTM-based architecture: embedding layer, LSTM, then multi-layer perceptron (MLP), with the simpler set-up of an embedding layer followed by a MLP of varying depth. Figure 5a depicts the performance of the LSTM model, with confidence intervals bootstrapped from a 10-fold cross-validation (CV), with the best performing MLP on the same 10 folds. Architectures were kept as similar as possible by using the same size hidden layers in the MLPs as in the LSTM model. We find the recurrent model significantly benefits model performance, indicating the utilisation of temporal information, even in the presence of thousands of variables. We also explore the effect of varying the number of percentile bins used to discretise continuous values in Fig. 5b. We find 10 bins offers the best performance of the three variants tested, providing the best trade-off between broad outlier categories and delineation of densely packed distributions.

For predicting inpatient mortality, the AUROC curve at 48 h after admission was 0.8463 (95% CI 0.8411–0.8513, bootstrapped from 10,000 samples of a 10-fold cross-validation). This was significantly stronger than traditional predictive models, with OASIS and SAPS II scores achieving 0.76 and 0.72 on a similar cohort^21^. After 48 h of patient data has been accumulated, an AUROC value of 0.85 means that there is a 85% chance our model will assign a higher probability to a randomly chosen patient destined to die rather than a randomly chosen patient destined to live. If clinical resource allocation were based on our model rather than SAPS II, approximately 18% more patients would be correctly prioritised.

### Predictive performance over time

**Figure 6:**
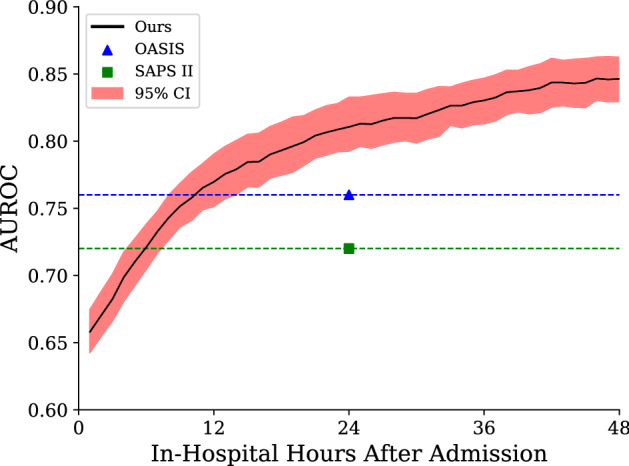
Comparison of our model’s dynamic predictive performance over the initial 48 h of the patient stay against OASIS and SAPS II calculated at 24 h (calculated in Harutyunyan et al.^21^). Confidence intervals were bootstrapped from the ten-fold cross-validation. Our model exceeds the performance of traditional scoring systems early in the patient stay, before continuing to improve during their stay.

In Fig. 6, we illustrate the performance of our model through time—that is, the predictive strength of our model after each hour of a patient’s stay. After the initial 6 h of a patient’s stay, our model has an AUROC of 0.72, equivalent with the overall performance of the SAPS II severity score. This is significant as SAPS relies on the entire first 24 h of patient data and cannot make a prediction before or after this point. The same limitation applies to OASIS, which is outperformed after 11 h of patient data. Indeed, after only 12 h, our model achieves an AUROC of 0.77 (95% CI 0.75–0.79), performance which is arguably strong enough to assist the actions and prioritisation of clinicians. After the initial increase in model AUROC, the rate of improvement was found to become more incremental. Although our model continues to accumulate useful information for mortality prediction through time, new information at later stages in a patient’s stay had less of an impact on prediction.

## Discussion

We present a deep learning model using the entire multivariable patient time-series, regardless of variable type or frequency, and without the need for variable selection or cleaning. Our method capitalises on the flexible nature of word embeddings from NLP^25,26,31^ and the success of RNNs in sequence analysis^18,19^, to greatly simplify the curation required to use existing EHR structures for prediction. Our model is dynamic and able to track predicted survival probability for optimal prediction timing as well as providing an estimate of prediction confidence. As the vast majority of current techniques cannot make predictions at arbitrary times^7,12^, our time-sensitive model could help: clinicians to assess overall patient trajectory, response to therapeutic interventions, guide optimal trials of intensive care, improve patient alerting, and contribute to optimally informed shared decision-making conversations. This formulation leads to a significant improvement over current ICU scoring systems in early patient outcome prediction when validating our method using the real-world ICU cohort of the MIMIC-III dataset^23^, with performance on-par with current literature on deep learning for EHRs^10,21,32,33^ despite no variable curation. Confident early prediction of low patient risk decreases the time needed for reassurance from traditional scoring systems and could be used as a guide for transfer from the ICU to a lower priority ward, potentially saving resources and staffing costs.

Unlike previous work, our model represents variables in a single large embedding space and is, to our knowledge, the first time all clinical variables from an EHR database have been represented in the same latent space. Therefore, our model is the first attempt to teach an ICU AI system to relate all EHR variables on the same basis—analogous to asking a healthcare provider to relate everything from demographic information to heart rate in an unbiased way. Complex machine learning techniques (and deep learning in particular) can be opaque, and this has been a criticism in the medical domain where decision-making must be transparent to be acceptable to both patients and clinicians. However, our model design inherently provides a degree of insight by ranking the relevance of clinical variables. As such, our results aid clinicians in focusing their attention across all clinical variables, help treatment decision making, and demonstrate surprisingly important factors in patient outcome prediction.

We created a processing and embedding technique that assimilated all discrete and continuous events in each patient’s EHR because we were interested in generalising the embedding mechanism demonstrated in^12^ to arbitrary EHR formats. Preservation of so many variable types allows our model to learn from a far broader range of ICU data than previous models. For instance, the model now assesses nursing notes at the same time as checking the most recent laboratory values; an experience much closer to that of a clinician. Our model also has the capacity to relate unusual or infrequently sampled events across time—insight that clinicians in the highly demanding setting of the ICU may struggle to appreciate. As the spread of EHR systems proliferates, larger datasets employing models with this type of flexible embedding could also lead to clinical and physiological insights beyond those of the current medical corpus. This superhuman comprehension of diverse data has already been demonstrated for image classification^34,35^ and the data dense setting provided by modern EHR systems is likely to prove equally fruitful.

**Figure 7:**
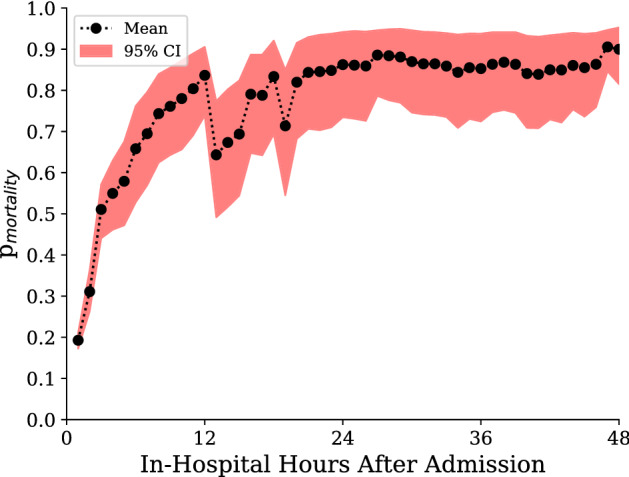
Dynamic probability of mortality after ICU admission for a patient who subsequently died during their stay in hospital. Event rank within each hour, event name, and event value (percentile) are shown for the first and last hour, as well as hours where there is a significant change in mortality likelihood.

### Case study

The combination of dynamic, individual mortality predictions with calibrated uncertainty provides a summary of the patient state which may be useful in automated alerting and clinician prioritisation. The time-sensitivity means that it may be used to track patient trajectory as well as evaluate response to therapy. The availability of ranked salient features is a step towards providing the interpretability needed to make such systems acceptable both to patients or patient advocates and clinicians in guiding individualised care. For example, Fig. 7 shows hourly predictions for a patient, a male of over 90 years of age on his second stay in the ICU after being discharged 2 months before, who died of congestive heart failure 65 h after admission. Our model made an initial prediction after 1 h of patient data, predicting that the patient had a 19.2% chance of mortality. This is already a significant deviation from the population average mortality rate of 13.2% and was based on the importance our model places on high blood pressure and high respiratory rate. Our model later indicates the patient deteriorating to over 50% risk of death at hour 3, due to worryingly low temperature and $\hbox {SpO}_2$. Assessment of our model’s predictions at this point in time would have already shown a very concerning trend in patient risk, potentially reinforcing clinician suspicions at the time. Subsequently, after 12 h, over 2 days before death occurred, our model gave the patient an 83.6% chance of death. By the time a traditional severity score such as OASIS or SAPS II could have been used to calculate patient mortality risk, our model had already indicated this patient was at incredibly high risk for several hours. At the end of our prediction window the likelihood of survival was less than 10%.

Figure 7 also highlights the limitations of our model. In the thirteenth hour of the patient’s stay, when there are only 6 readings taken, it is unclear why the model prioritises Service CME and Allergy 1 No Known drug allergies. As the model is broad enough to process all clinical variables, it will also rank many superfluous records that could affect prediction. Also, due to the limitations of the task at hand—prediction of mortality at 48 hours—our model may struggle to generalise further into the patient stay. For example, lab assays change over time and could change normal ranges, but such changes are typically small. Another limitation of the study is the inherent bias introduced when obtaining quantiles from specific hospital cohorts. This renders our model subject to both hospital-based and demographic-based bias. Moreover, the principle concern with maximally flexible models that can intake all forms of input, including clinician notes, is that models will learn to extract trivial identifiers of impending outcomes. This could include discharge notes being indicative of survival, or clinician comments on patient outcomes being directly used for prediction. In our experiments we found events such as recording the code status instructions for cpr, assigning consent to a next of kin, and even visitation by the priest to all be highly indicative of mortality. By employing clearly interpretable models, such as our ranking system, a clinician could ignore such tautologies and decide whether to consider the implications of our model’s next most important events.

### Conclusion

We believe our model to be the first that performs no variable selection or data curation while allowing for erroneous values. Recent work^12,36^ with EHR data, has focused on scalability and streamlining the transition between widespread data formats and model inputs. Our model goes one step further by allowing for all types of readings to be used as inputs and assessed for any correlation with patient outcomes. Furthermore, when employing our pipeline, there is no unnecessary removal of patients during secondary analysis of EHR data. We present a deep learning model which can generate time-sensitive mortality probability estimates as a summary measure of patient state with calibrated confidence estimates for an individual patient at any arbitrary time. The model is able to assimilate all the data available without the need for cleaning. Unlike previous ICU prediction models, we treat all variables in the same manner, without the need for feature engineering, using a single large embedding space. Even without data curation, for early prediction, we achieve very strong performance across the cohort. Our approach is a natural way to handle the complex structure of ICU data, providing a compact summary of patient state over time while making the salient features available to clinicians for potential guidance.

## Data Availability

The MIMIC-III database is part of restricted-access clinical data maintained by PhysioNet (MIMIC-II, MIMIC-III, eICU Collaborative Research Database) and is available, subject to a formal research request, from the MIT Laboratory for Computational Physiology and their collaborating research groups https://mimic.physionet.org/gettingstarted/access/. The code used for these experiments is available at https://github.com/jacobdeasy/flexible-ehr.
